# Editorial: Talent Identification and Development in Sports Performance

**DOI:** 10.3389/fspor.2021.729167

**Published:** 2021-11-24

**Authors:** Nuno Leite, Alberto Lorenzo Calvo, Sean Cumming, Bruno Gonçalves, Julio Calleja-Gonzalez

**Affiliations:** ^1^Research Centre in Sports Sciences, Health Sciences and Human Development (CIDESD), Vila Real, Portugal; ^2^Departamento de Ciências do Desporto Exercício e Saúde, ECVA, Universidade de Trás-os-Montes e Alto Douro, Vila Real, Portugal; ^3^Departamento de Deportes, Facultad de Ciencias de la Actividad Física y del Deporte—Instituto Nacional de Educación Física, Universidad Politécnica de Madrid, Madrid, Spain; ^4^Department for Health, University of Bath, Bath, United Kingdom; ^5^Departamento de Desporto e Saúde, Escola de Saúde e Desenvolvimento Humano, Universidade de Évora, Évora, Portugal; ^6^Comprehensive Health Research Centre (CHRC), Universidade de Évora, Évora, Portugal; ^7^Portugal Football School, Portuguese Football Federation, Oeiras, Portugal; ^8^Department of Physical Education and Sport, Faculty of Education and Sport, University of the Basque Country, Vitoria-Gasteiz, Spain

**Keywords:** maturation, growth, youth, technology, performance analysis

Talent identification and development have become increasingly relevant in sports performance (Sarmento et al., 2018), especially in the last 20 years. A significant body of scientific research discusses longitudinal and non-linear talent identification and development processes, the qualities that underpin elite performance in sport, and how coaches could facilitate talented athletes' development through the sports system (Baker et al., 2020). Yet it can be argued that the continued interest in talent identification and development reflects the persistently low predictive value of applied and theoretical talent identification models (Till and Baker, 2020).

Finding, recruiting, and retaining talent is a global challenge, and it is especially relevant for sports clubs and national federations that often see potential assets escape due to self-system inefficiencies (Koz et al., 2012). In point of fact, original research selected for this topic confirmed that talent development programs should make conscious decisions about their selection strategies as it can affect their success (Kalén et al.; Dugdale et al.).

Globally, this Research Topic contributed to successfully collate applied research presenting some of the latest evidence of the use of technologies for measuring and analyzing talent. Other methodological advances have drawn on non-linear approaches, as well as the importance of socio-cultural determinents (Reverberi et al.; Coutinho et al.) playing out in increasingly complex and multidimensional environments (Höner et al.; Ribeiro-Junior et al.). In addition to these topics, nsightful contributions may be found on the debate about the importance of quality of the early engagement experience (Sweeney et al.), paralympic sport (Dehghansai et al.), and/or a balanced analysis of sport and health-related indicators (Bjørndal et al.).

Despite growing evidence of core influencing factors on talent ID and development, many coaches and stakeholders continue to fail to consider adequately important factors such as relative age (de la Rubia et al.), growth, maturation, training age, or to distinguish among these constructs effectively (Lloyd et al., 2014; Arede et al., 2021). The potential of unequal policy and practice implications of biased models that prioritize the athlete's current performance and therefore obviate their somatic and maturational development are also discussed (Leyhr et al.; Arede et al.; Arede et al.).

Under the vast majority of contemporary sports systems, participants are categorized into annual age groups to reduce the developmental differences during childhood and adolescence. Although age groups are ideal for matching players on attributes that follow age, they are not without their limitations. Specifically, children of the same age can vary in skeletal maturity, an established index of maturation in youth, by as much as 5–6 years (Saward et al.). Most sports federations select young athletes based on current competition results rather than development potential. This means that many of these talent selection processes fail to integrate essential indicators when assessing young talent (Romann, 2020). Given these shortcomings, several sports clubs, academies and federations have committed to adding new strategies such as bio-banding or testing indicators in talent selection, the maturational status or the peak height velocity (Arede et al., 2021). Although there is a growing map of scientific research investigating the independent effects of anthropometric, physiological, psychological (Schmid et al.; Taylor et al.) or contextual factors (Uehara et al.) among others to the talent identification process, relatively few of them, have considered the potential interactions among these variables, especially, the between contextual and socioeconomic factors on nurturing talent regardless of age-related issues or communities' size (Leite et al.).

New comprehensive research proposals are emerging (Bonney et al.) to improve overall analysis of entire processes impacting talent ID and development. Ideas like “birthday-banding” (Kelly et al., 2020), the “transfer talent” (Vaeyens et al., 2009) “process talent transfer” (Pion et al., 2020) or “specialized sampling” (Sieghartsleitner et al., 2018), will not only help to obtain better results but also lose (or retain) fewer athletes throughout the way. Very few talent development processes have an efficiency rate > 30% (e.g., see the works of Boccia et al., 2020, 2021 in athletics, or Koz et al., 2012, in professional sports). It is precisely this last point that should also capture our attention in greater depth. Most research in this area focuses on the successful athlete, ignoring that athletes will not be successful. Perhaps it is time to look the other way, better understand why some athletes fail to achieve these performance levels and consequently improve the process further and lose fewer athletes (Williams and MacNamara, 2020).

Understanding the interaction between nature and nurture is critical for better understanding talent identification and development (Williams and MacNamara, 2020). Indeed, the growing complexity of processes impacting talentID are highly unpredictable and it will never be possible to consider this phenomenon as an exact science. Furthermore, this complexity illuminates distinctly individual and personal processes contextualized to each participant's situation, and therefore not reproducible in other individuals. In this regard, progress in research, specifically in social epigenetics (Ahmetov and Fedotovskaya, 2015; Pickering et al., 2019), should offer relevant contributions in the coming years. As noted, the process of predicting which athletes are most likely to succeed (i.e., international, national or regional competition) will always encompass risks and mistakes (Born et al.). Mistakes when judging *potential* vs. *performance* must be considered the likely consequence of a process of own self-regulation that excludes talent from one sport while unconsciously taking it to another one (Collins et al., 2014).

Recognizing a certain level of scientific inconsistency that typically is associated with talent identification and development in sport (Baker et al., 2020), there is an overabundance of research employing cross-sectional designs and descriptive analysis methods on this topic (Jackson and Comber, 2020). In this sense, this issued privileged (a) longitudinal (Post et al.; Saward et al.) and prospective studies (Höner et al.), (b) non-mainstream sports (Roberts et al.), (c) inclusive approaches (Dehghansai et al.), (d) and the transference from this research to other countries and continents (Uehara et al.).

The lack of consensus in talent definition can lead to extreme positions and heterogeneous positioning among practitioners, coaches, scouts, sports scientists, athletes, and scientific community members (Kravariti and Johnston, 2019). While disagreement can foster scholarly debate and, consequently, lead to a better understanding of a particular phenomenon, it can also serve as a barrier for application. In addition, it should be noted that talent is subject to continuing evolution, via (i) future paradigms and social challenges that are transforming sport and physical activity, understood as a public health challenge and a phenomenon of socialization, (ii) of the rapidly rising diversity of media exposure of sports or, or (iii) exceptional technological advancements and the growing sophistication of methods used in the sports industry. Epstein (2004) compared former exceptional athletes like Jesse Owens or Roger Banister and their best performances with nowadays features. Changing technologies, changing genes, and a changing mindset may ultimately help explaining why the athletes are getting stronger, faster, bolder, and better than ever. As an example, focus on the recent feature by the Kenyan marathon runner Eliud Kipchoge, who made history in athletics last year by finishing for the first time, the total distance of a marathon under 2 h (Hoogkamer et al., 2019).

The recent technological and processing techniques revolution and the ongoing methodological refinement may have led to a scientific research paradigm change explored in the current Research Topic (Bedir and Erhan). To what extent all these evolving technologies have affected talent identification and development, and the selection process still requires much broader and deeper investigation. Thus, all this body of scientific knowledge may be incorporated into the performance analysis scope, instigating new questions about the dynamical parameters of performance and potentially opening new windows of opportunity. In particular, one aspect on which we clearly must work, advance, and improves the relationship between scientific research and the practical world (Stricker and Goldfried, 2019). Therefore, the criticism of this gap between science and practice is certainly understood as fair (Sandbakk, 2018; Haugen, 2019).

Globally, talentID Research Topic combined empirical research and theoretical models that contribute to advancing knowledge related to sports performance, specifically concerning its role in talent identification and proper development. The collection of these scientific articles included in the Research Topic debated and provided a holistic contribution on how this knowledge should be applied, both in organizational structures (sports clubs, youth academies, national federations, and research centers) and in strategic policies responsible for promoting and fostering long-term performance programmes, youth participation, and personal development. This is the main reason why the editors consider that this editorial article highlighted the need to understand the obligations and needs of the practitioners, to bridge the gap between science and practice (Collins et al., 2018) and provide a helpful guide to be effectively translated to daily practice in sports.

The development of human and technological resources, mostly visible in several sports sciences (from psychology to nutrition, from physiology to biomechanics, through motor learning or pedagogy), plays a key role and responds to the question posed by Epstein (2004). The evolution of each sports speciality is a consequence of all the technological, regulatory and knowledge advances, which will undoubtedly provoke the appearance or understanding of new models of successful athletes. Advances in data processing and analysis and the capacity to collect such data contribute to our understanding of what talent is and the process of identifying and developing it. In this sense, big data is playing an epicentral role in helping scientists better understand players behaviors, such as dynamic positioning in their natural environments (Gonçalves et al., 2018) and within their in-game actions (Fernandez and Bornn, 2018). Alongside, advanced processing techniques have accompanied this journey, such as machine learning and deep learning (Cust et al., 2019; Musa et al., 2020), data mining (Dubois et al., 2020), artificial intelligence (Claudino et al., 2019), or training programs based on virtual reality and virtual augmented interaction (Michalski et al., 2019).

Indeed, as the advancement of knowledge across multiple disciplines allows for greater depth of understanding about talentID, it also helps applied knowledge of impacts at a practical level in the athlete's day-to-day life. The athlete's development process can be converted into a more controlled and less random process, and we can advance in improving the efficiency of all the athlete's development processes and programs (not only at the level of performance but also the level of health—physical and mental—of the athlete). To do this, inevitably, there must first be a clear consensus among researchers to establish who is considered a talented athlete. To advance knowledge in this field, it is necessary to start from a broad consensus among the scientific community members on this topic.

## Author Contributions

All authors contributed equally to manuscript revision, read, and approved the submitted version.

## Conflict of Interest

The authors declare that the research was conducted in the absence of any commercial or financial relationships that could be construed as a potential conflict of interest.

## Publisher's Note

All claims expressed in this article are solely those of the authors and do not necessarily represent those of their affiliated organizations, or those of the publisher, the editors and the reviewers. Any product that may be evaluated in this article, or claim that may be made by its manufacturer, is not guaranteed or endorsed by the publisher.

## References

[B1] Ahmetov I. Fedotovskaya O. (2015). Current progress in sports genomics. Adv. Clin. Chem. 70, 247–314. 10.1016/bs.acc.03003 26231489

[B2] Arede J. Cumming S. Johnson D. Leite N. (2021). The effects of maturity matched and un-matched opposition on physical performance and spatial exploration behavior during youth basketball matches. PLoS ONE 16:e0249739. 10.1371/journal.pone.0249739 33831106 PMC8031392

[B3] Baker J. Wilson S. Johnston K. Dehghansa N. Koenigsberg A. Vegt de N.. (2020). Talent research in sport 1990–2018: a scoping review. Front. Psychol. 1:607710. 10.3389/fpsyg.2020.607710 33324305 PMC7723867

[B4] Boccia G. Cardinale M. Brustio P. R. (2020). World-class sprinters' careers: early success does not guarantee success at adult age. Int. J. Sports Physiol. Perform. 16, 367–374. 10.1123/ijspp.2020-0090 33296871

[B5] Boccia G. Cardinale M. Brustio P. R. (2021). Performance progression of elite jumpers: early performances do not predict later success. Scand. J. Med. Sci. Sports 31, 132–139. 10.1111/sms.13819 32881090

[B6] Claudino J. G. Capanema D. O. Souza de Serrão, T. V. Machado Pereira J. C. Nassis A. C. G. P. (2019). Current approaches to the use of artificial intelligence for injury risk assessment and performance prediction in team sports: a systematic review. Sports Med. Open 5:28. 10.1186/s40798-019-0202-3 31270636 PMC6609928

[B7] Collins D. MacNamara A. Cruickshank A. (2018). Research and practice in talent identification and development—some thoughts on the state of play. J. Appl. Sport Psychol. 31, 340–351. 10.1080/10413200.2018.1475430

[B8] Collins R. Collins D. MacNamara A. Jones M. I. (2014). Change of plans: an evaluation of the effectiveness and underlying mechanisms of successful talent transfer. J. Sports Sci. 32, 1621–1630. 10.1080/02640414.2014.908324 24814474

[B9] Cust E. E. Sweeting A. J. Ball K. Robertson S. (2019). Machine and deep learning for sport-specific movement recognition: a systematic review of model development and performance. J. Sports Sci. 37, 568–600. 10.1080/02640414.2018.1521769 30307362

[B10] Dubois R. Bru N. Paillard T. Le Cunuder A. Lyons M. Maurelli O.. (2020). Rugby game performances and weekly workload: Using of data mining process to enter in the complexity. PLoS ONE 15:e0228107. 10.1371/journal.pone.0228107 31995600 PMC6988915

[B11] Epstein D. (2004). The Sports Gene: Talent, Practice and the Truth About Success. London: Yellow Jersey Press.

[B12] Fernandez J. Bornn L. (2018). Wide Open Spaces: A statistical technique for measuring space creation in professional soccer, in Proceedings of the MIT Sloan Sports Analytics Conference, Boston, MA.

[B13] Gonçalves B. Coutinho D. Travassos B. Folgado H. Caixinha P. Sampaio J. (2018). Speed synchronization, physical workload and match-to-match performance variation of elite football players. PLoS ONE 13:e0200019. 10.1371/journal.pone.0200019 30040849 PMC6057640

[B14] Haugen T. (2019). Key success factors for merging sport science and best practice. Int. J. Sports Physiol. Perform. 15:297. 10.1123/ijspp.2019-0940 31883503

[B15] Hoogkamer W. Snyder K. L. Arellano C. J. (2019). Reflecting on Eliud Kipchoge's marathon world record: an update to our model of cooperative drafting and its potential for a sub-2-hour performance. Sports Med. 49, 167–170. 10.1007/s40279-019-01056-2 30671908

[B16] Jackson R. C. Comber G. J. (2020). Hill on a mountaintop: a longitudinal and cross-sectional analysis of the relative age effect in competitive youth football. J. Sports Sci. 38, 1352–1358. 10.1080/02640414.2019.1706830 31916503

[B17] Kelly A. L. Jackson D. T. Taylor J. J. Jeffreys M. A. Turnnidge J. (2020). Birthday-Banding as a strategy to moderate the relative age effect: a case study into the England squash talent pathway. Front. Sports Active Living 2:573890. 10.3389/fspor.2020.573890 33345136 PMC7739587

[B18] Koz D. Fraser-Thomas J. Baker J. (2012). Accuracy of professional sports drafts in predicting career potential. Scand. J. Med. Sci. Sports 22, e64–e69. 10.1111/j.1600-0838.2011.01408.x 22092367

[B19] Kravariti F. Johnston K. (2019). Talent management: a critical literature review and research agenda for public sector human resource management. Public Management Review 22, 75–95. 10.1080/14719037.2019.1638439

[B20] Lloyd R. Oliver J. Faigenbaum A. Myer G. Ste Croix D. E. (2014). Chronological age vs. biological maturation: implications for exercise programming in youth. J. Strength Condit. Res. 28, 1454–1464. 10.1519/JSC.0000000000000391 24476778

[B21] Michalski S. C. Szpak A. Loetscher T. (2019). Using virtual environments to improve real-world motor skills in sports: a systematic review. Front. Psychol. 10:2159. 10.3389/fpsyg.2019.02159 31620063 PMC6763583

[B22] Musa R. M. Majeed A. P. P. A. Kosni N. A. Abdullah M. R. (2020). Machine Learning in Team Sports: Performance Analysis and Talent Identification in Beach Soccer and Sepak-takraw. Springer.

[B23] Pickering C. Kiely J. Grgic J. Lucia A. Del Coso J. (2019). Can genetic testing identify talent for sport? Genes 10:972. 10.3390/genes10120972 31779250 PMC6969917

[B24] Pion J. Teunissen J. W. Ter Welle S. Spruijtenburg G. Faber I. R. Lenoir M. (2020). Chapter 13: how similarities and differences between sports lead to talent transfer: a process approach, in Talent Identification and Development in Sport: International Perspectives 2nd edition, eds J. Baker, S. Cobley, and J. Schorer (London: Routledge). pp 184–196. 10.4324/9781003049111-13

[B25] Romann M. (2020). Improving Talent Identification Through Analysis and Consideration of Biological and Relative Age. 10.13140/RG.2.2.13062.80961

[B26] Sandbakk Ø. (2018). Let's close the gap between research and practice to discover new land together! *Int. J. Sports Physiol. Perform*.13:961. 10.1123/ijspp.2018-0550 30189759

[B27] Sarmento H. Anguera M. T. Pereira A. Araújo D. (2018). Talent identification and development in male football: a systematic review. Sports Med. 48, 907–931. 10.1007/s40279-017-0851-7 29299878

[B28] Sieghartsleitner R. Zuber C. Zibung M. Conzelmann A. (2018). The early specialised bird catches the worm!–a specialised sampling model in the development of football talents. Front. Psychol. 9:188. 10.3389/fpsyg.2018.00188 29515500 PMC5826374

[B29] Stricker G. Goldfried M. R. (2019). The gap between science and practice: A conversation. Psychotherapy 56, 149–155. 10.1037/pst0000220 30816763

[B30] Till K. Baker J. (2020). Challenges and [possible] solutions to optimising talent identification and development in sport. Front. Psychol. 11:664. 10.3389/fpsyg.2020.00664 32351427 PMC7174680

[B31] Vaeyens R. Güllich A. Warr C. R. Philippaerts R. (2009). Talent identification and promotion programmes of Olympic athletes. J. Sports Sci. 27, 1367–1380. 10.1080/02640410903110974 19787538

[B32] Williams G. MacNamara Á. (2020). I Didn't Make It, but…: deselected athletes' experiences of the talent development pathway. Front. Sports Active Living 2:24. 10.3389/fspor.2020.00024 33345018 PMC7739725

